# The genome sequence of the Atlantic horse mackerel, *Trachurus trachurus* (Linnaeus 1758)

**DOI:** 10.12688/wellcomeopenres.17813.1

**Published:** 2022-03-31

**Authors:** Martin Genner, Rupert Collins, Wellcome Sanger Institute Tree of Life programme, Wellcome Sanger Institute Scientific Operations: DNA Pipelines collective, Tree of Life Core Informatics collective, Darwin Tree of Life Consortium

**Affiliations:** 1 University of Bristol, Bristol, UK

**Keywords:** Trachurus trachurus, Atlantic horse mackerel, genome sequence, chromosomal

## Abstract

We present a genome assembly from an individual *Trachurus trachurus *(the Atlantic horse mackerel; Chordata; Actinopteri; Carangiformes; Carangidae). The genome sequence is 801 megabases in span. The majority of the assembly, 98.68%, is scaffolded into 24 chromosomal pseudomolecules. Gene annotation of this assembly on Ensembl has identified 25,797 protein coding genes.

## Species taxonomy

Eukaryota; Metazoa; Chordata; Craniata; Vertebrata; Euteleostomi; Actinopterygii; Neopterygii; Teleostei; Neoteleostei; Acanthomorphata; Carangaria; Carangiformes; Carangidae; Trachurus; *Trachurus trachurus* (Linnaeus, 1758) (NCBI txid:36212).

## Background

The Atlantic horse mackerel *Trachurus trachurus* (Linnaeus, 1758), also known as European horse mackerel or common scad, is northern Europe’s only resident representative of the Carangidae, a ray-finned fish family that includes the jacks, pompanos and trevallies. *Trachurus trachurus* is a benthopelagic shoaling species and is typically found at depths of less than 200 m. The species has a broad distribution, including Iceland, Northeast Atlantic continental shelf waters, the Mediterranean, and north-western African coastal waters at least as far as Ghana ( Healey *et al.*, 2020). Atlantic horse mackerel are targeted by commercial fisheries using trawls, purse seines and long-lines. Major fished stocks are managed regionally. Those in Northeast Atlantic continental shelf waters are separated into a southern stock (Atlantic waters of the Iberian Peninsula), a western stock (shelf-edge seas from Bay of Biscay to the Norwegian coast, including spawning grounds of the Celtic Sea), and a North Sea stock (central and southern North Sea, including the Skagerrak and Kattegat) ( ICES, 2019). Total landings of 140,000 metric tonnes were reported in 2018, down from catches of over 450,000 metric tonnes in the mid 1990s. On the basis of declining abundance over sections of the species range, it has been listed as Vulnerable by the International Union for the Conservation of Nature ( Smith-Vaniz *et al.*, 2015).

## Genome sequence report

The genome was sequenced from a single *T. trachurus* of unknown sex collected from Southampton Water, off the coast of Hampshire, UK. A total of 105-fold coverage in Pacific Biosciences single-molecule long reads (N50 23 kb) and 64-fold coverage in 10X Genomics read clouds (from molecules with an estimated N50 of 22 kb) were generated. Primary assembly contigs were scaffolded with chromosome conformation Hi-C data. Manual assembly curation corrected 141 missing/misjoins and removed 43 haplotypic duplications, reducing the scaffold number by 22.14%, increasing the scaffold N50 by 19.37% and decreasing the assembly length by 1.57%.

**Figure 1.**
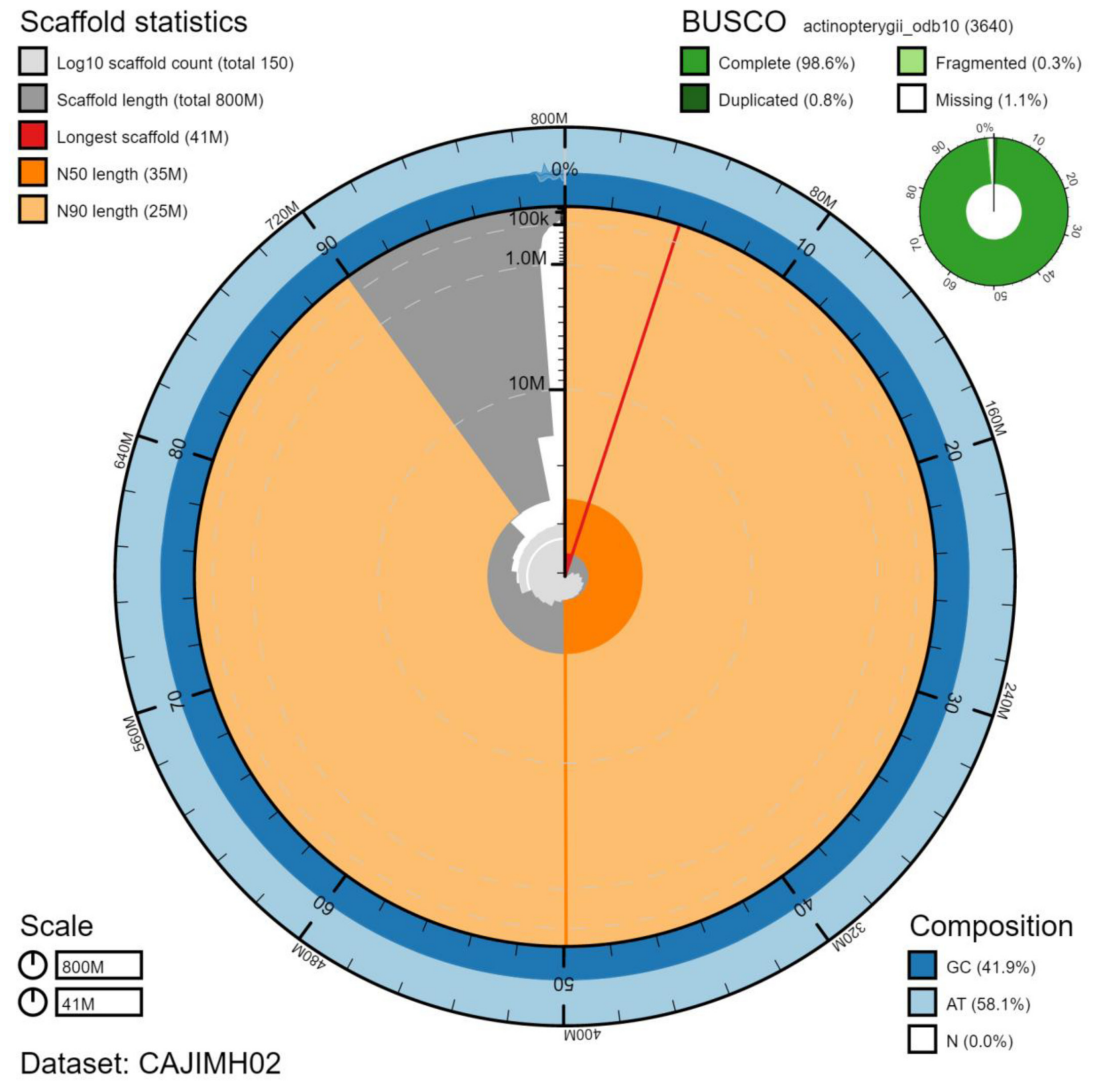
Genome assembly of *Trachurus trachurus*, fTraTra1.2: metrics. The BlobToolKit Snailplot shows N50 metrics and BUSCO gene completeness. The main plot is divided into 1,000 size-ordered bins around the circumference with each bin representing 0.1% of the 801,243,942 bp assembly. The distribution of scaffold lengths is shown in dark grey with the plot radius scaled to the longest scaffold present in the assembly (40,754,244 bp, shown in red). Orange and pale-orange arcs show the N50 and N90 scaffold lengths (35,447,499 and 25,462,409 bp), respectively. The pale grey spiral shows the cumulative scaffold count on a log scale with white scale lines showing successive orders of magnitude. The blue and pale-blue area around the outside of the plot shows the distribution of GC, AT and N percentages in the same bins as the inner plot. A summary of complete, fragmented, duplicated and missing BUSCO genes in the actinopterygii_odb10 set is shown in the top right. An interactive version of this figure is available at https://blobtoolkit.genomehubs.org/view/fTraTra1.2/dataset/CAJIMH02/snail.

The final assembly has a total length of 801 Mb in 152 sequence scaffolds with a scaffold N50 of 35.4 Mb ( Table 1). The majority, 98.68%, of the assembly sequence was assigned to 24 chromosomal-level scaffolds, representing 24 autosomes (numbered by synteny to *Oryzias latipes* (Japanese medaka); GCF_002234675.1) ( Figure 1– Figure 4; Table 2). The assembly has a BUSCO v5.1.2 ( Manni *et al.*, 2021) completeness of 98.6% using the actinopterygii_odb10 reference set. While not fully phased, the assembly deposited is of one haplotype. Contigs corresponding to the second haplotype have also been deposited.

**Table 1.** Genome data for *Trachurus trachurus*, fTraTra1.2.

*Project accession data*	
Assembly identifier	fTraTra1.2
Species	*Trachurus trachurus*
Specimen	BMNH 2021.3.19.1; fTraTra1
NCBI taxonomy ID	NCBI:txid36212
BioProject	PRJEB42240
BioSample ID	SAMEA7524396
Isolate information	Muscle
*Raw data accessions*	
PacificBiosciences SEQUEL II	ERR6445210
10X Genomics Illumina	ERX5643309, ERX5643310, ERX5693250, ERX5693251
Hi-C Illumina	ERR6054366-ERR6054368
*Genome assembly*	
Assembly accession	GCA_905171665.2
*Accession of alternate haplotype*	GCA_905171655.2
Span (Mb)	801
Number of contigs	374
Contig N50 length (Mb)	6.49
Number of scaffolds	152
Scaffold N50 length (Mb)	35.45
Longest scaffold (Mb)	40.75
BUSCO * genome score	C:98.6%[S:97.8%,D:0.8%], F:0.3%,M:1.1%,n:3640
Genome annotation **	
Number of protein-coding genes	25,797
Average length of protein-coding gene (bp)	1,811
Average number of exons per gene	12
Average exon size (bp)	178
Average intron size (bp)	1,755

*BUSCO scores based on the actinopterygii_odb10 BUSCO set using v5.1.2. C= complete [S= single copy, D=duplicated], F=fragmented, M=missing, n=number of orthologues in comparison. A full set of BUSCO scores is available at https://blobtoolkit.genomehubs.org/view/fTraTra1.2/dataset/CAJIMH02/busco.

**Genome annotation provided for assembly fTraTra1.1.

**Figure 2.**
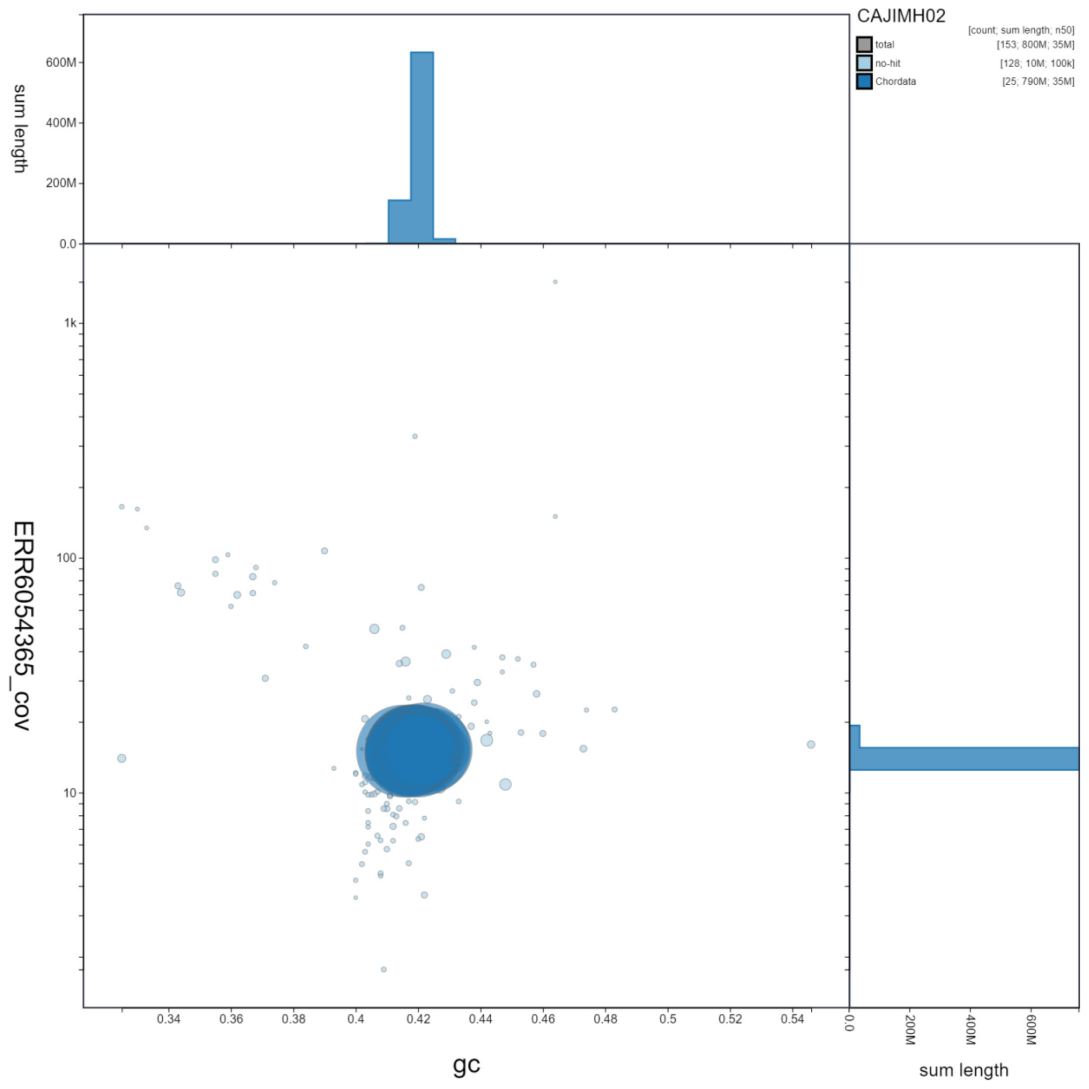
Genome assembly of Trachurus trachurus, fTraTra1.2: GC coverage. BlobToolKit GC-coverage plot. Scaffolds are coloured by phylum. Circles are sized in proportion to scaffold length. Histograms show the distribution of scaffold length sum along each axis. An interactive version of this figure is available at https://blobtoolkit.genomehubs.org/view/fTraTra1.2/dataset/CAJIMH02/blob.

**Figure 3.**
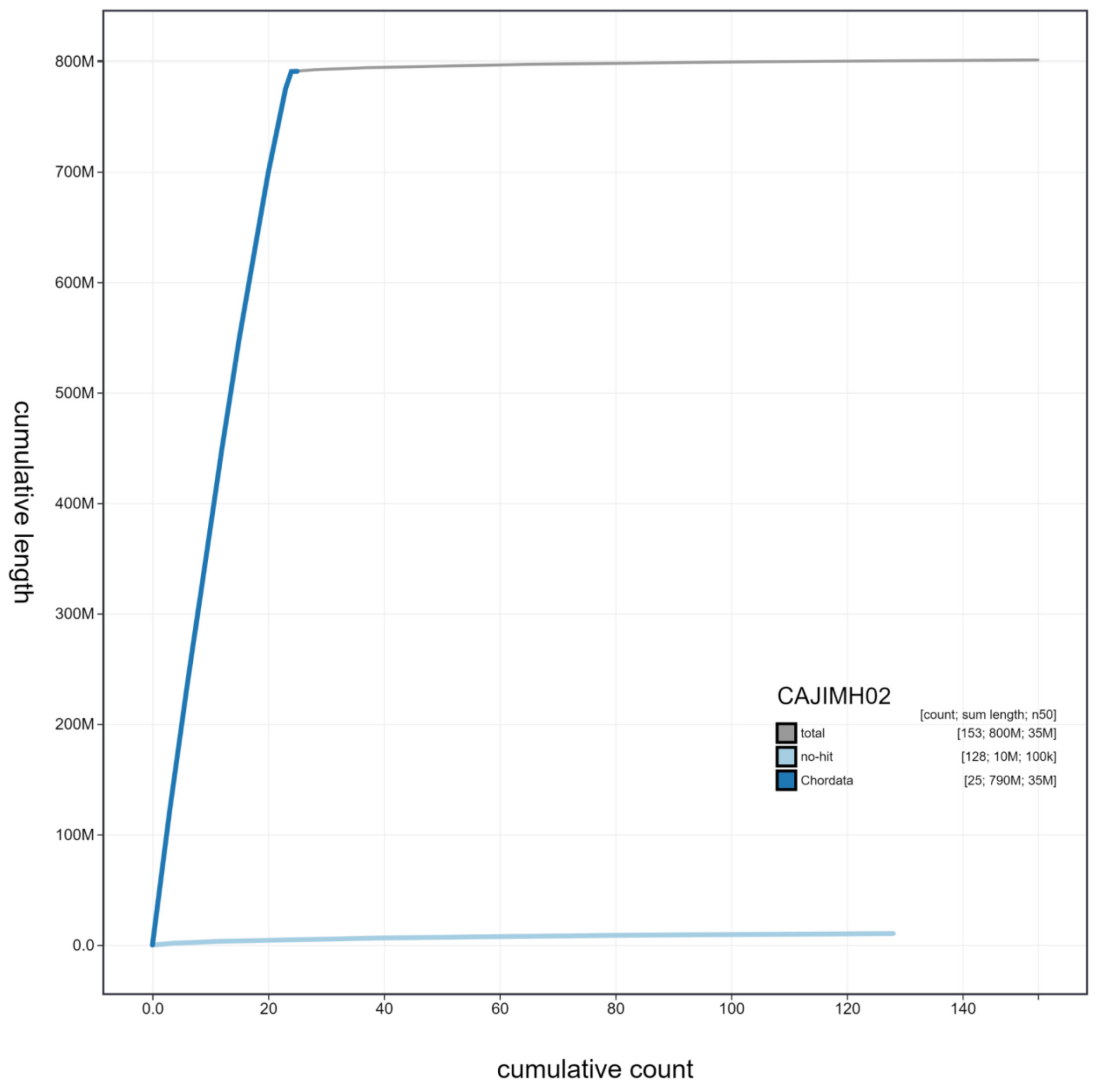
Genome assembly of *Trachurus trachurus*, fTraTra1.2: cumulative sequence. BlobToolKit cumulative sequence plot. The grey line shows cumulative length for all scaffolds. Coloured lines show cumulative lengths of scaffolds assigned to each phylum using the buscogenes taxrule. An interactive version of this figure is available at https://blobtoolkit.genomehubs.org/view/fTraTra1.2/dataset/CAJIMH02/cumulative.

**Figure 4.**
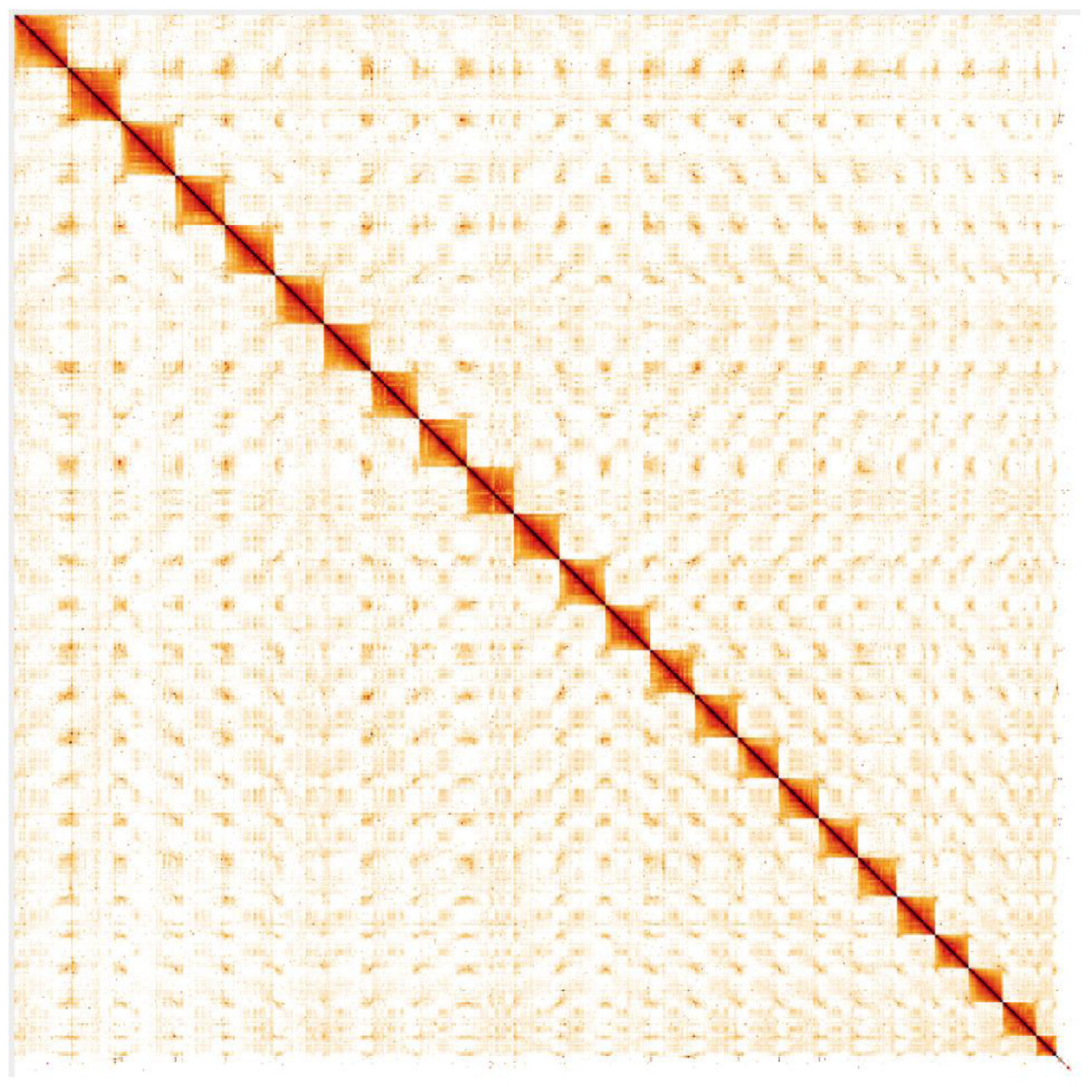
Genome assembly of *Trachurus trachurus*, fTraTra1.2: Hi-C contact map. Hi-C contact map of the fTraTra1.2 assembly, visualised in HiGlass. Chromosomes are given in order of size from top to bottom and left to right. The interactive Hi-C map can be viewed here.

**Table 2.** Chromosomal pseudomolecules in the genome assembly of *Trachurus trachurus*, fTraTra1.2.

INSDC accession	Chromosome	Size (Mb)	GC%
LR991628.1	1	40.75	41.8
LR991651.1	2	15.47	42.7
LR991630.1	3	40.66	41.5
LR991635.1	4	36.09	41.9
LR991633.1	5	36.89	41.7
LR991634.1	6	36.28	41.9
LR991638.1	7	35.45	42.0
LR991642.1	8	33.05	42.4
LR991632.1	9	37.40	41.9
LR991631.1	10	38.57	41.8
LR991641.1	11	33.39	42.1
LR991645.1	12	29.58	41.6
LR991629.1	13	40.70	42.2
LR991640.1	14	33.71	41.8
LR991643.1	15	31.05	41.9
LR991639.1	16	35.01	41.9
LR991637.1	17	35.71	42.0
LR991646.1	18	29.25	42.0
LR991650.1	19	25.33	42.2
LR991648.1	20	25.46	42.2
LR991636.1	21	35.83	41.7
LR991644.1	22	30.60	42.2
LR991649.1	23	25.35	42.1
LR991647.1	24	29.13	42.0
LR991652.1	MT	0.02	46.5
-	Unplaced	10.50	31.6

## Gene annotation

The Ensembl gene annotation system ( Aken *et al.*, 2016) was used to generate annotation for the *T. trachurus* assembly (GCA_905171665.1). Annotation was created primarily through alignment of transcriptomic data to the genome, with gap filling via protein to-genome alignments of a select set of vertebrate proteins from UniProt ( UniProt Consortium, 2019) and coordinate mapping of GENCODE ( Frankish *et al.*, 2019) mouse reference annotations via a pairwise whole genome alignment. The resulting Ensembl annotation includes 60,310 transcripts assigned to 25,797 coding and 2,264 non-coding genes ( Trachurus trachurus - Ensembl Rapid Release).

## Methods

### Sample acquisition, DNA extraction and sequencing

A single *T. trachurus* of unknown sex was collected in January 2017 from near Marchwood Power Station in Southampton Water, off the coast of Hampshire, UK (latitude 50.901563, longitude -1.440836) by Rupert Collins as part of the SeaDNA project.

DNA was extracted using an agarose plug extraction from muscle tissue following the Bionano Prep Animal Tissue DNA Isolation Soft Tissue Protocol. Pacific Biosciences CLR long read and 10X Genomics read cloud sequencing libraries were constructed according to the manufacturers’ instructions. Sequencing was performed by the Scientific Operations core at the Wellcome Sanger Institute on Pacific Biosciences SEQUEL II and Illumina HiSeq X instruments. Hi-C data were generated from muscle tissue using the Arima Hi-C kit and sequenced using a HiSeq X instrument.

### Genome assembly

Assembly was carried out following the Vertebrate Genome Project pipeline ( Rhie *et al.*, 2020) with Falcon-unzip ( Chin *et al.*, 2016). Haplotypic duplication was identified and removed with purge_dups ( Guan *et al.*, 2020) and a first round of scaffolding carried out with 10X Genomics read clouds using scaff10x. Scaffolding with Hi-C data ( Rao *et al.*, 2014) was carried out with SALSA2 ( Ghurye *et al.*, 2019). The Hi-C scaffolded assembly was polished with arrow using the PacBio data, then polished with the 10X Genomics Illumina data by aligning to the assembly with longranger align, calling variants with freebayes ( Garrison & Marth, 2012) and applying homozygous non-reference edits using bcftools consensus. Two rounds of the Illumina polishing were applied. The mitochondrial genome was assembled using the mitoVGP pipeline ( Formenti *et al.*, 2021). The assembly was checked for contamination and corrected using the gEVAL system ( Chow *et al.*, 2016) as described previously ( Howe *et al.*, 2021). Manual curation ( Howe *et al.*, 2021) was performed using gEVAL, HiGlass ( Kerpedjiev *et al.*, 2018) and Pretext. The genome was analysed and BUSCO scores generated using BlobToolKit ( Challis *et al.*, 2020). Table 3 contains a list of all software tool versions used, where appropriate.

**Table 3.** Software tools used.

Software tool	Version	Source
Falcon_unzip	1.4.2	Chin *et al.*, 2016
purge_dups	1.0.1	Guan *et al.*, 2020
scaff10x	4.1	https://github.com/wtsi-hpag/Scaff10X
SALSA2	2.2	Ghurye *et al.*, 2019
arrow	GCpp-1.9.0	https://github.com/PacificBiosciences/GenomicConsensus
freebayes	1.3.1-17-gaa2ace8	Garrison & Marth, 2012
mitoVGP	2.2	Formenti *et al.*, 2021
gEVAL	N/A	Chow *et al.*, 2016
HiGlass	1.11.6	Kerpedjiev *et al.*, 2018
PretextView	0.1.x	https://github.com/wtsi-hpag/PretextView
BlobToolKit	2.6.4	Challis *et al.*, 2020

## Data availability

European Nucleotide Archive: Trachurus trachurus (Atlantic horse mackerel). Accession number PRJEB42240; https://identifiers.org/ena.embl/PRJEB42240.

The genome sequence is released openly for reuse. The *T. trachurus* genome sequencing initiative is part of the Darwin Tree of Life (DToL) project and Vertebrate Genome Project (VGP). The specimen has been frozen and deposited with the Natural History Museum, London under registration number BMNH 2021.3.19.1, where it will remain accessible to the research community for posterity. All raw sequence data and the assembly have been deposited in INSDC databases. Raw data and assembly accession identifiers are reported in Table 1.

## Author information

Members of the Wellcome Sanger Institute Tree of Life programme are listed here: https://doi.org/10.5281/zenodo.6125027.

Members of Wellcome Sanger Institute Scientific Operations: DNA Pipelines collective are listed here: https://doi.org/10.5281/zenodo.5746904.

Members of the Tree of Life Core Informatics collective are listed here: https://doi.org/10.5281/zenodo.6125046.

Members of the Darwin Tree of Life Consortium are listed here: https://doi.org/10.5281/zenodo.5638618.

## References

[ref-1] Aken BL Ayling S Barrell D : The Ensembl Gene Annotation System. *Database (Oxford).* 2016;2016:baw093. 10.1093/database/baw093 27337980 PMC4919035

[ref-2] Challis R Richards E Rajan J : BlobToolKit--Interactive Quality Assessment of Genome Assemblies. *G3 (Bethesda).* 2020;10(4):1361–1374. 10.1534/g3.119.400908 32071071 PMC7144090

[ref-3] Chin CS Peluso P Sedlazeck FJ : Phased Diploid Genome Assembly with Single-Molecule Real-Time Sequencing. *Nat Methods.* 2016;13(12):1050–54. 10.1038/nmeth.4035 27749838 PMC5503144

[ref-4] Chow W Brugger K Caccamo M : gEVAL — a Web-Based Browser for Evaluating Genome Assemblies. *Bioinformatics.* 2016;32(16):2508–10. 10.1093/bioinformatics/btw159 27153597 PMC4978925

[ref-5] Formenti G Rhie A Balacco J : Complete Vertebrate Mitogenomes Reveal Widespread Repeats and Gene Duplications. *Genome Biol.* 2021;22(1):120. 10.1186/s13059-021-02336-9 33910595 PMC8082918

[ref-6] Frankish A Diekhans M Ferreira AM : GENCODE Reference Annotation for the Human and Mouse Genomes. *Nucleic Acids Res.* 2019;47(D1):D766–D773. 10.1093/nar/gky955 30357393 PMC6323946

[ref-7] Garrison E Marth G : Haplotype-Based Variant Detection from Short-Read Sequencing. arXiv: 1207.3907.2012. Reference Source

[ref-8] Ghurye J Rhie A Walenz BP : Integrating Hi-C Links with Assembly Graphs for Chromosome-Scale Assembly. *PLoS Comput Biol.* 2019;15(8):e1007273. 10.1371/journal.pcbi.1007273 31433799 PMC6719893

[ref-9] Guan D McCarthy SA Wood J : Identifying and Removing Haplotypic Duplication in Primary Genome Assemblies. *Bioinformatics.* 2020;36(9):2896–2898. 10.1093/bioinformatics/btaa025 31971576 PMC7203741

[ref-10] Healey AJE Farthing MW Nunoo FKE : Genetic Analysis Provides Insights into Species Distribution and Population Structure in East Atlantic Horse Mackerel ( *Trachurus Trachurus* and *T. Capensis*). *J Fish Biol.* 2020;96(3):795–805. 10.1111/jfb.14276 32031244 PMC7079130

[ref-11] Howe K Chow W Collins J : Significantly Improving the Quality of Genome Assemblies through Curation. *Gigascience.* 2021;10(1):giaa153. 10.1093/gigascience/giaa153 33420778 PMC7794651

[ref-12] ICES: Working Group on Widely Distributed Stocks(WGWIDE). *ICES Scientific Reports*.2019;1(36). Reference Source

[ref-20] Kerpedjiev P Abdennur N Lekschas F : HiGlass: web-based visual exploration and analysis of genome interaction maps. *Genome Biol.* 2018;19(1):125. 10.1186/s13059-018-1486-1 30143029 PMC6109259

[ref-13] Manni M Berkeley MR Seppey M : BUSCO Update: Novel and Streamlined Workflows along with Broader and Deeper Phylogenetic Coverage for Scoring of Eukaryotic, Prokaryotic, and Viral Genomes. *Mol Biol Evol.* 2021;38(10):4647–54. 10.1093/molbev/msab199 34320186 PMC8476166

[ref-14] Rao SSP Huntley MH Durand NC : A 3D Map of the Human Genome at Kilobase Resolution Reveals Principles of Chromatin Looping. *Cell.* 2014;159(7):1665–80. 10.1016/j.cell.2014.11.021 25497547 PMC5635824

[ref-15] Rhie A McCarthy SA Fedrigo O : Towards Complete and Error-Free Genome Assemblies of All Vertebrate Species. *bioRxiv.* 2020;2020.05.22.110833. 10.1101/2020.05.22.110833v1 PMC8081667 33911273

[ref-16] Smith-Vaniz WF Sidibe A Nunoo F : Trachurus Trachurus. IUCN Red List of Threatened Species.2015. 10.2305/IUCN.UK.2015-4.RLTS.T198647A43157137.en

[ref-17] UniProt Consortium: UniProt: A Worldwide Hub of Protein Knowledge. *Nucleic Acids Res.* 2019;47(D1):D506–15. 10.1093/nar/gky1049 30395287 PMC6323992

