# Supplementary material for: The genome sequence of the Atlantic horse mackerel, Trachurus trachurus (Linnaeus 1758)
Source: Wellcome Open Res. 2022 Mar 31;7:118. [Version 1] doi: 10.12688/wellcomeopenres.17813.1 (PMC9975430; doi:10.12688/wellcomeopenres.17813.1)
Supplement: Peer review history

# Peer review: Reviewer response for version 1

**DOI:** 10.21956/wellcomeopenres.19721.r53698

**Published:** 2023-01-10

**Authors:** Adelaide Rhodes

**Affiliations:** 1 Tufts University, Medford, MA, USA

This genome report is an important contribution to the development of genome information an important fisheries stock from the family Carangidae, as the second published chromosome level assembly within the family and the third chromosome level assembly for the order Carangiformes. The paper does a comprehensive and complete job of laying out the methodology and results of the genome assembly. It is also written in a FAIR (findable, accessible, interoperable and reusable) format which will assist future researchers who may be responsible for managing the stocks. The final assembly has a total length of 801 Mb, found in 24 autosomes and 128 unplaced scaffolds with a scaffold N50 of 35.4 Mb. The prediction of 24 autosomal chromosomes is consistent with the only other chromosome level genome published for the the Carangidae family, *Seriola aureovittata*, which is smaller than the *T. trachurus* genome by approximately 151 Mb.

 Only one minor error was detected, the link to the Hi-C interactive map in Figure 4 did not work at the time of this review. It would be nice to have a fixed link in regards to examining the data in more detail.

 Another small issue is that the genetic material came from a single sample of unknown sex. Sex determination in fish as a whole is complicated, it would be helpful in future genome studies to find some confirmation of the sex of the sample if possible through gonadal cannulation or biopsy.

 In addition the genome report finds after generating the 24 autosomes based on synteny to *Oryzias latipes* (Japanese medaka) that 128 scaffolds of N50 104,180 and length 10.5 Mb fell into the unplaced sequence. It would be interesting to determine if some polishing of the genome or the pseudo-alternative haplotype could resolve these unplaced scaffolds, perhaps by choosing a different fish model for syntenic comparison.

 An alternative haplotype was generated containing 1,049 scaffolds with total length 797 Mb and an N50 of 1.6 Mb. It would be helpful, in this reviewer's opinion, to provide a few more details about the alignments of the alternate locus reference sequences to the main chromosome sequences in the assembly to put these alternative loci into the context of the reference genome.

 A few more comments on the significance of this genome report:

 The horse mackerel is not a true "mackerel", which represents another important fisheries stock. It may be helpful, therefore, to point out that true mackerels are in the family Scombridae which holds many highly migratory species with unique challenges and adaptations. In contrast, the horse mackerel has two significant populations. The Northern stock of the horse mackerel spawns in the North Sea and heads back to Northern waters, whereas the Western stock spawns in the Bay of Biscay and heads west as the fish mature.

 The family Carangidae contains 30 genera and approximately 152 species, which have very few whole genome assemblies as a group. At the time of this review, in National Center for Biotechnoloy Information (NCBI), National Library of Medicine (NLM), hosted by the National Institutes of Health (NIH), USA (https://www.ncbi.nlm.nih.gov/data-hub/taxonomy/1489907/), there are currently fifteen published Carangidae genomes other than the two from this genome report. Nine genomes are from the genus *Seriola*, which contains the amberjacks; two are from the genus *Caranx* (giant trevally and bluefin trevally) and three are from the genus from *Trachinotaus, *all three are from the golden pompano *Trachinotus ovatus*. *Seriola aureovittata* ( the great amberjack, synonymous with *Seriola lalandi*) is the only other species in the family Carangidae with a chromosome level assembly. Within the order Carangiformes, only one other fish, the live sharksucker *Echeneis naucrates *has a chromosome level assembly.

 Overall, this genome report is a significant contribution which will help in the management of important fisheries stocks that are under pressure from climate change, habitat reduction and increasing economic demand for food from the oceans.

Are sufficient details of methods and materials provided to allow replication by others?

Yes

Is the rationale for creating the dataset(s) clearly described?

Yes

Are the datasets clearly presented in a useable and accessible format?

Partly

Are the protocols appropriate and is the work technically sound?

Yes

Reviewer Expertise:

Marine biology, Bioinformatics, Whole genome assemblies of non-model organisms, Metagenomics.

I confirm that I have read this submission and believe that I have an appropriate level of expertise to confirm that it is of an acceptable scientific standard.

# Peer review: Reviewer response for version 1

**DOI:** 10.21956/wellcomeopenres.19721.r53697

**Published:** 2023-01-03

**Authors:** Josephine Paris

**Affiliations:** 1 Department of Health, Life and Environmental Sciences, University of L'Aquila, Coppito, Italy

This Data Note article presents a chromosome-level assembly of *Trachurus trachurus* using long-read, short-read and Hi-C data for assembly. Being a commercially-important species, and the only resident member of the Carangidae family, the rationale for genomically enabling this species is very clear. The protocol for assembly follows the well-established VGP pipeline and the annotation is performed by Ensembl using the latest available proteomic analysis. Regarding the annotation, I miss some more detail on where the transcriptomic data is from (i.e. publicly available or generated in this project?) and also from which tissues the data are derived from (e.g. a broader range of tissues = a better chance of capturing all the protein-coding genes). I suggest this information is added to the article. Methods are clear and replicable. The genome, and its raw data, are available via the appropriate channels (i.e. NCBI / ENA), as well as the annotation via Ensembl's Rapid Release.

Are sufficient details of methods and materials provided to allow replication by others?

Yes

Is the rationale for creating the dataset(s) clearly described?

Yes

Are the datasets clearly presented in a useable and accessible format?

Yes

Are the protocols appropriate and is the work technically sound?

Yes

Reviewer Expertise:

Population genomics, genome assembly, transcriptomics

I confirm that I have read this submission and believe that I have an appropriate level of expertise to confirm that it is of an acceptable scientific standard.

# Peer review: Reviewer response for version 1

**DOI:** 10.21956/wellcomeopenres.19721.r49622

**Published:** 2022-04-21

**Authors:** Luís Filipe C. Castro

**Affiliations:** 1 CIIMAR-Interdisciplinary Centre of Marine and Environmental Research, U. Porto-University of Porto, Porto, Portugal

This genome provides a valuable resource in the context of teleost species that represent a fishery resource. The lack of a fully phased genome is not a significant problem. This will provide an opportunity for comparative genomics approaches with the ever growing number of high-quality genomes currently available for this range of taxa.

Are sufficient details of methods and materials provided to allow replication by others?

Yes

Is the rationale for creating the dataset(s) clearly described?

Yes

Are the datasets clearly presented in a useable and accessible format?

Yes

Are the protocols appropriate and is the work technically sound?

Yes

Reviewer Expertise:

Comparative Genomics

I confirm that I have read this submission and believe that I have an appropriate level of expertise to confirm that it is of an acceptable scientific standard.
